# Enhancing Behavioral Health Competencies: Interprofessional Collaboration Between Social Work and Medicine

**DOI:** 10.1093/geroni/igab046.1881

**Published:** 2021-12-17

**Authors:** Bronwyn Keefe

**Affiliations:** Boston University, Boston, Massachusetts, United States

## Abstract

This presentation will describe the creation and findings from an interprofessional curriculum in behavioral health developed by social work faculty for medical students. Training in behavioral health is needed more than ever during a time of increased isolation and fear during the COVID pandemic. Older adults with untreated behavioral health concerns are a vulnerable population, which can result in negative effects, including emotional distress, reduced physical health, increased mortality, and suicide (IOM, 2012). Healthcare is increasingly complex with a need to focus on the physical, social, and behavioral aspects of daily living, and providers are realizing the importance of interprofessional collaboration. Towards that aim, I created a module for 4th year medical students in mental health and older adults, which is now part of their medical education curriculum. I will present outcomes in: (1) satisfaction; (2) acquired knowledge and skills (post-test); (3) application of knowledge and skills (pre-post competency assessment and comfort around asking about depression); and (4) patient outcomes (frequency of depression screening and number of referrals to social worker). Feedback from the 143 medical students is positive with 95% strongly agreeing or agreeing that this expanded their knowledge and understanding in mental health issues among older adults. At baseline, 17% of medical students were moderately to very comfortable in asking questions on the GDS compared to 42% at post-assessment. After completing the course, almost 25% of medical students made a referral to social work during their rotation. This collaboration resulted in curriculum that is both rigorous and impactful.

